# Empathy for Pain from Adolescence through Adulthood: An Event-Related Brain Potential Study

**DOI:** 10.3389/fpsyg.2012.00501

**Published:** 2012-11-26

**Authors:** Nathalie Mella, Joseph Studer, Anne-Laure Gilet, Gisela Labouvie-Vief

**Affiliations:** ^1^University of GenevaGeneva, Switzerland; ^2^Alcohol Treatment Center, Lausanne University Hospital CHUVLausanne, Switzerland; ^3^Division of Old Age Psychiatry, Lausanne University Hospital CHUVLausanne, Switzerland; ^4^Laboratoire de Psychologie des Pays de la Loire (LPPL EA 4638), University of NantesNantes, France

**Keywords:** adolescence, empathy, emotion regulation, pain perception

## Abstract

Affective and cognitive empathy are traditionally differentiated, the affective component being concerned with resonating with another’s emotional state, whereas the cognitive component reflects regulation of the resulting distress and understanding of another’s mental states (see Decety and Jackson, 2004 for a review). Adolescence is a critical period for the development of cognitive control processes necessary to regulate affective processes: it is only in young adulthood that these control processes achieve maturity (Steinberg, 2005). Thus, one should expect adolescents to show greater automatic empathy than young adults. The present study aimed at exploring the neural correlates of affective (automatic) and cognitive empathy for pain from adolescence to young adulthood. With this aim, Event Related Potentials (ERPs) were recorded in 32 participants (aged 11–39) in a task designed to dissociate these components. ERPs results showed an early automatic fronto-central response to pain (that was not modulated by task demand) and a late parietal response to painful stimuli modulated by attention to pain cues. Adolescents exhibited earlier automatic responses to painful situations than young adults did and showed greater activity in the late cognitive component even when viewing neutral stimuli. Results are discussed in the context of the development of regulatory abilities during adolescence.

## Introduction

Empathy is a complex emotion that plays a critical role in promoting successful social relationships (Batson and Shaw, 1991). The ability to empathize is likely to be particularly important during early adolescence when maintaining peer relationships becomes central to well-being. Empathic skills have for example been shown to be involved in the good perception of socially relevant cues to interpret a message (van den Brink et al., 2012). While there have been a great number of studies exploring empathic abilities in adolescents with psychiatric disorders, such as autism (Demurie et al., 2011) or schizophrenia (e.g., Shamay-Tsoory et al., 2007), or in adolescents showing aggressive conduct (see Lovett and Sheffield, 2007 for a review), little is known about the development of empathic skills in normal adolescence, and still less concerning its neural underpinnings. The present research aims at exploring age-related differences in the neural response of empathy for pain assessed in adults and adolescents using electroencephalography (EEG).

Empathy refers to the ability to share and understand others’ emotion or feeling (Decety and Lamm, 2006). Experiencing empathy relies on the integration of two components: a phylogenetically and ontogenetically early emotional contagion system and a more advanced cognitive system that allows self-regulation and elaboration of the situation (Preston and De Waal, 2002; Decety and Jackson, 2004). The former system entails an automatic affective resonance with the others’ emotional experience thought to be mediated by shared neural representations (Gallese, 2003; Gallese et al., 2004). Resonance between other and self may lead to personal distress (i.e., feelings of discomfort and anxiety; Lamm et al., 2007a). In contrast, mature forms of empathy require that one understand the others’ need and can trigger sympathy. The primary affective response needs therefore to be modulated by self-regulation processes, beginning with basic forms of self-other distinction and leading to more advanced forms of perspective-taking abilities. Eventually, mature empathy is characterized by elaborated conscious forms of emotion regulation. All of these conscious regulatory processes tend to be costly in terms of the investment of effort and should depend on the maturation of cortico-limbic connections.

In line with this theoretical argument, developmental research has widely demonstrated this progression from more automatic forms of empathy to ones that are better regulated. For example babies show emotional contagion in response to the distress of another individual without being able to separate their own and the other’s distress (Thompson, 1987). Self-other differentiation begins later in childhood and develops through adolescence (Hoffman, 1985; Harter, 1998). Recent evidence also suggests a continued development of the ability to understand other’s emotions and mental states between adolescence and adulthood (Blakemore, 2008). Adolescence is also marked by heightened emotional reactivity and immature top-down prefrontal control systems (Steinberg, 2005; Hare et al., 2008). Using fMRI, Hare et al. (2008) showed that adolescents displayed heightened activity in subcortical emotional processing systems and less functional fronto-limbic connectivity when viewing emotional pictures. According to Steinberg (2005) this dissociation between heightened emotional arousability and the late maturation of brain regions involved in the cognitive abilities necessary to down-regulate emotions renders adolescents more vulnerable emotionally. Since brain networks mediating cognitive empathy are not fully mature in early adolescents, they should therefore be less efficient in down-regulating the primary affective response in the experience of empathy.

Neuroimaging research in the field of empathy has mostly been interested in empathy for pain because of the universality and automaticity of the affective response elicited when witnessing another’s pain. A number of studies have shown that the cognitive and affective components do rely on distinct neural networks (see Decety and Meyer, 2008, for a review). For example, studies have reported an overlap between the neural regions underlying the personal experience of pain (affective component) and those activated while observing another expressing pain. More specifically, activation is consistently observed in the anterior insula and anterior medial cingulate cortex (aMCC; Morrison et al., 2004; Singer et al., 2004; Lamm et al., 2007a, 2011; Singer and Lamm, 2009), and to a lesser extent in the somatosensory cortex and the cerebellum (see Lamm et al., 2011 and Singer and Lamm, 2009, for a review). In contrast, the cognitive components of empathy have been shown to rely on a network of regions that are associated with emotion regulation, such as prefrontal dorsolateral and median prefrontal cortices (Lamm et al., 2007b) or with mentalizing, such as the temporo-parietal junction, the temporal poles, and the precuneus/posterior cingulate cortex (PCC; Jackson et al., 2006). FMRI studies of empathy for pain hence provide arguments in support of the assumption that empathy is a two-component process. Investigating the temporal dynamics of perception of pain with the ERP method, Fan and collaborators (Fan and Han, 2008; Han et al., 2008) dissociated in an elegant way the affective component from the cognitive component of empathy by manipulating attention to pain cues. The authors reported a dissociation between an early automatic emotional sharing component (double fronto-central negativity, N110 and N340) and a late cognitive component (centro-parietal LPP; Fan and Han, 2008; Han et al., 2008). Some ERPs studies have shown that these two components are modulated by several factors such as medical expertise (Decety et al., 2010), gender (Han et al., 2008), or cognitive strategies (Sheng and Han, 2012). From a developmental perspective, one should also expect a modulation of these automatic and cognitive aspects of emotional processing (Labouvie-Vief et al., 2010). In the present research, we used the same paradigm than Han and collaborators to test age-related differences between adolescents and adults’ ERPs reflecting affective and cognitive empathy for pain. We hypothesized that adolescents will exhibit stronger automatic affective responses when witnessing another in a painful situation than adults. Moreover, regarding the assumed immaturity of brain networks involved in down-regulation and mentalizing abilities in adolescence, we also expected age-related differences in the cognitive component of empathy.

## Materials and Methods

### Participants

Sixteen adolescents (mean age: 13.1 years) and 16 adults (mean age: 33.8 years) with no history of neurological or psychiatric disorder volunteered for this study. All participants were female. Fluid abilities, crystallized abilities, and depression were assessed in all participants. One adult and two adolescents had to be excluded from data analyses because of excessive artifact in the EEG signal. The participants’ characteristics are detailed in Table 1. The study was approved by the local ethical committee and all participants gave their informed consent.

**Table 1:** **Participants’ characteristics**.

	Adolescents (*N* = 16)	Adults (*N* = 16)
Age *M* (SD), age spread	13.1^a^ (1.13), 11–14.6	33.8^b^ (4.69), 26.1–39.2
Depression *M* (SD)	13.1 (6.98)	12.4 (10.53)
Speed of processing *M* (SD)	64.06^a^ (7.76)	84.94^b^ (11.23)
Vocabulary *M* (SD)	35 (6.22)	37.25 (2.74)

*^a,b^Means with different supscripts differed significantly (*p* < 0.001) between age groups*.

*Depression was measured by the French version of the CES-D (Fuhrer and Rouillon, 1989). Speed of processing was measured by the subtest digit symbol substitution of the WISC (Wechsler, 2003) for the adolescents and the WAIS (Wechsler, 1997) for the adults. Vocabulary was measured by the subtest Vocabulary (WISC, Wechsler, 2003) for the adolescents and the Mill Hill (Deltour, 1993) for the adults*.

### Materials

All participants completed a measure of dispositional empathy, the French version of the Empathy Quotient (EQ; Berthoz et al., 2008), which allows distinguishing cognitive aspects of empathy from affective ones. But in order to ensure that our measure was well adapted to younger participants, adolescents also completed the Basic Empathic Scale (BES, French version; D’Ambrosio et al., 2009), which is specifically designed for adolescents and also taps cognitive and affective aspects of empathy. In addition, participants completed the Stroop Colour task (Stroop, 1935) in order to provide a measure of individual ability of inhibitory control.

Experimental stimuli were presented using E-prime 1.2 on a DELL computer (Schneider et al., 2002). The stimuli were the same as those used by Fan and collaborators (Fan and Han, 2008; Han et al., 2008) and consisted in 40 digital color pictures showing one hand or two hands in painful and neutral situations. The pictures were shot from the first-person perspective and described accidents that may happen in everyday life, such as a hand trapped in a door or cut by scissors. Twenty pictures showed hands in painful situation (one hand in eight painful pictures and two hands in 12 painful pictures). Each painful picture was matched with a neutral picture that showed one or two hands in situations that, although similar in contexts, did not imply any pain.

Subjective measures regarding the stimuli were assessed using the Face Pain Scale-Revised (FPS-R; Bieri et al., 1990), which contains six faces showing neutral to extremely painful expression. Both the intensity of pain supposedly felt by the person on the picture (others’ pain) and the intensity of personal discomfort felt by the participants (self-unpleasantness) were measured.

### Procedure and design

Participants completed the behavioral part of the experiment first, the order of tests, and questionnaires being pseudo-randomly assigned to participants. The EEG session was completed within 2 weeks after the behavioral part. At the beginning of the EEG session, participants were equipped with a 64 electrodes cap and comfortably installed on a chair, in a quiet room dedicated to EEG recording.

The task consisted eight blocks of 80 trials in which pictures were presented for 200 ms, which is very fast and allows controlling for attention in order to dissociate automatic from cognitive responses to pain. In half of the blocks, participants had to judge whether the situation was painful or not (attention to pain) and in the four other blocks, they had to decide whether there were one or two hands on the picture (attention withdrawn from painful indices). The stimulus was immediately followed by a fixation cross lasting 1500 ms, during which participants gave their response with their right and left fingers on a response-pad. The assigned response-buttons were counterbalanced across participants. After 1500 ms, the color of the cross changed during a varying interval between 300 and 450 ms to indicate a new trial was beginning.

After the task, pictures were presented for 2000 ms and participants were asked to evaluate (1) the intensity of the pain supposedly felt by the model on the picture (other’s pain evaluations) and (2) the degree of their self-unpleasantness.

### ERP data recording and analysis

The EEG was recorded from 64 scalp electrodes that were mounted on an electrocap in accordance to the extended 10–20 system. EEG signal was continuously recorded at a 2048 Hz sampling rate using a Biosemi system (Amsterdam, Netherlands). Electrodes were referenced offline using average signal (Picton et al., 2000). It is noteworthy that the use of average reference does not allow age group comparisons, as they certainly differ in many aspects that can affect that average potential (e.g., the maturation of cortical tissue). Accordingly, higher ERPs amplitudes are traditionally observed with adolescents than with adults (e.g., Segalowitz et al., 2010). However, this should affect neither main effects of Pain and Task, nor interactions with Age group. EEG signal was then resampled at 256 Hz, filtered (high-pass: 0.4 Hz; low-pass 40 Hz, notch: 50 Hz). Eye blinks and vertical eye movements were then removed using an Independent Component Analysis (ICA) with Brain Vision Analyzer Software (Brain Products GmBH). The ERPs were then computed in each condition separately with an epoch beginning 200 ms before stimulus onset (baseline) and continuing for 1000 ms. ERPs were averaged for each electrode, each experimental condition, and each subject. Lastly, grand averages were computed for each electrode, each experimental condition, and each age group.

Statistical analyses were conducted at electrodes selected from the frontal-central (FCz, FC3–FC4), and parietal (Pz, P3–P4) regions. ANOVAs were run with Age as a between-subjects factor and Electrode position, Task and Pain as within-subjects factors. When needed, Tukey tests were used for *post hoc* analyses.

## Results

### Behavioral results

#### Response times and response accuracies

The mean RTs and response accuracies in each condition are shown in Table 2. ANOVAs conducted on RTs showed significant main effects of Task *F*(1,30) = 177.28, *p* < 0.001, η^2^ = 0.86 and Pain, *F*(1,30) = 18.77, *p* < 0.001, η^2^ = 0.38. The Task × Pain interaction was also significant, *F*(1,30) = 19.54, *p* < 0.001, η^2^ = 0.39. *Post hoc* analyses indicate that painful pictures were associated to significantly lower RTs when participant attended to pain, while there was no effect of Pain when participants attended to the number of hands.

**Table 2:** **Mean response times (RT in ms) and accuracy (mean percentage of correct responses, CR) by condition and age group**.

	Adolescents		Adults	
	Counting	Pain judgment	Counting	Pain judgment
CR neutral stimuli	91.23 (6.61)	80.39 (10.84)	98.18 (1.56)	89.53 (7.12)
CR painful stimuli	90.76 (5.87)	78.31 (12.22)	98.16 (1.28)	88.65 (4.45)
RT neutral stimuli	644.46 (100.52)	866.57 (101.33)	603.70 (69.73)	835.12 (108.50)
RT painful stimuli	646.23 (90.15)	823.69 (95.58)	600.53 (73.64)	773.29 (109.78)

*Standard deviations are given in brackets*.

ANOVAs conducted on response accuracies showed a significant main effect of Age, *F*(1,30) = 24.33, *p* < 0.001, η^2^ = 0.45, indicating that adolescents had less correct responses than adults. Main effect of Task was also significant, *F*(1,30) = 122.77, *p* < 0.001, η^2^ = 0.80: participants were more accurate when counting hands than when judging pain. Neither other main effects nor interactions were significant.

#### Self-assessed dispositional empathy (BES and EQ)

To verify that the EQ was also appropriated to assess dispositional empathy in our adolescent sample, correlations were computed between scores obtained for the BES, specifically designed for adolescents, and scores obtained with the EQ. Overall, correlations were high and significant. Global scores correlated at *r* = 0.64 (*p* < 0.01), and both scores assessing cognitive empathy and affective empathy were significantly correlated (*r* = 0.60, *p* < 0.05 and *r* = 0.63, *p* < 0.01, respectively). Therefore, only scores assessed by the EQ were analyzed.

*T* tests computed on the global scores and on both cognitive and affective scores did not reveal any significant age-related difference (all *p*s > 0.05).

#### Measure of inhibitory control (stroop-color word test)

Interference scores (*I*) were computed as follows: *I* = CW – *predicted* CW; where *predicted* CW = (*C* × *W*)/(*C* + *W*); *C* = score for the color denomination, *W* = score for the word denomination, and CW = score for the denomination of the colored words. A negative score then indicates a high level of interference.

*T* tests conducted on interference scores did not show any significant difference between adults’ scores and adolescents’ scores, *t*(30) = −0.42, *p* = 0.674, even if adolescents displayed lower mean scores (*M* = 1.77, SD = 6.47) than adults (*M* = 2.17, SD = 6.16).

### ERP results

**Figure 1:**
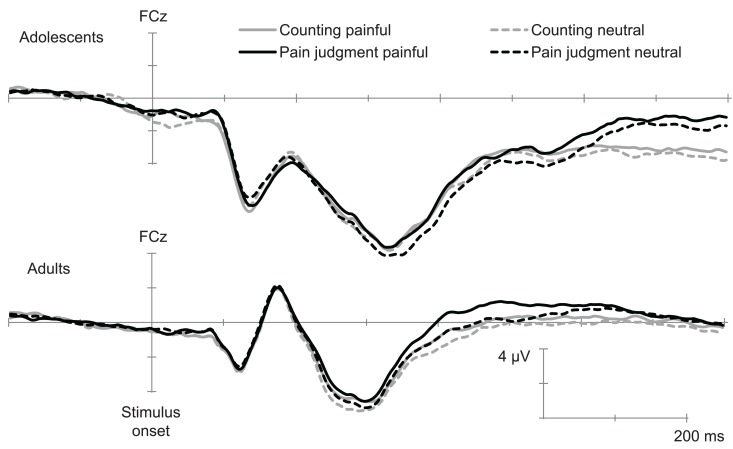
**Temporal course of early ERPs elicited by painful and non-painful pictures in the pain judgment task and in the counting task (Grand mean of 15 adults and 14 adolescents)**. This illustration shows the N110 and N340 recorded over FCz.

**Figure 2:**
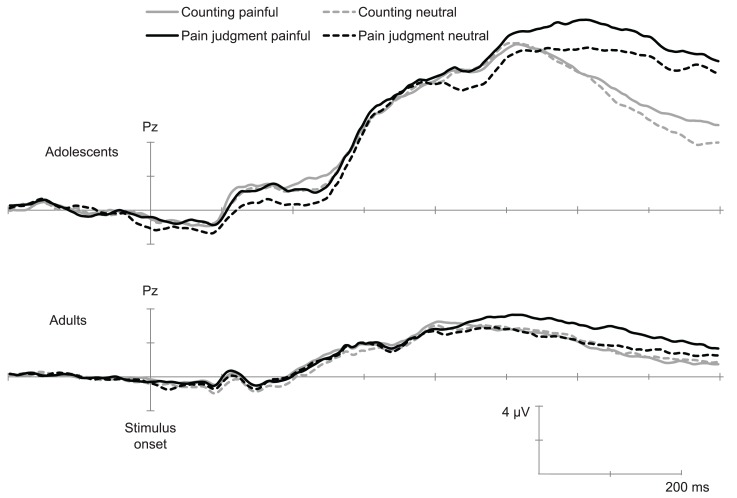
**Temporal course of late ERPs elicited by painful and non-painful pictures in the pain judgment task and in the counting task (Grand mean of 15 adults and 14 adolescents)**. This illustration shows the LPP recorded over Pz.

Inspection of the ERPs (grand means) showed, in all conditions and in agreement with previous studies using this paradigm (e.g., Han et al., 2008) a negative component between 90 and 130 ms (N110) over the frontal–central area, followed by a positive deflection and another negative wave peaking at 340 ms (N340). This wave was followed by a late positive potential between 360 and 800 ms (LPP) with the maximum amplitude over the parietal area. ERPs over the occipito-temporal area were characterized with a positivity wave between 80 and 140 ms (P1), a negative wave between 140 and 200 ms (N170), and a positive wave between 200 and 450 ms (P320). Figures 1 and 2 show the temporal course of each ERP component.

Previous studies having used this paradigm suggest that the N110, N340, and LPP are particularly related to pain judgment (Fan and Han, 2008; Han et al., 2008; Decety et al., 2010). Analyses were therefore conducted over the peak of amplitude on these components (see Table 3 for mean and standard deviation of each component’s amplitude).

**Table 3:** **Mean amplitudes (SD) of the N110, N340, and LPP, in each experimental condition, for adolescents and adults**.

	N110	N340	LPP
**ADOLESCENTS**			
Counting-*P*	−6.75 (0.56)	−8.52 (0.71)	10.44 (0.67)
Counting-*N*	−6.51 (0.54)	−8.64 (0.61)	10.42 (0.63)
Pain judgment-*P*	−6.63 (0.52)	−8.43 (0.69)	12.27 (0.83)
Pain judgment-*N*	−6.15 (0.57)	−8.85 (0.69)	10.94 (0.75)
**ADULTS**			
Counting-*P*	−2.64 (0.52)	−4.18 (0.66)	4.19 (0.63)
Counting-*N*	−2.77 (0.50)	−4.52 (0.57)	4.16 (0.59)
Pain judgment-*P*	−2.71 (0.48)	−3.92 (0.64)	4.46 (0.78)
Pain judgment-*N*	−2.58 (0.53)	−4.16 (0.65)	3.85 (0.70)

*Counting-*P*, Counting task with painful stimuli; Counting-*N*, Counting task with neutral stimuli; Pain Judgment-*P*, Pain judgment task with painful stimuli; Pain Judgment-*N*, Pain judgment task with neutral stimuli*.

*For the N110 and N340, mean amplitudes are presented as a mean of FCZ, FC3, and FC4*.

*For the LPP, mean amplitudes are presented over Pz only, as the Pain × Task interaction was only significant over Pz*.

#### N110

The ANOVA conducted over fronto-central electrodes (FCz, FC3–FC4) indicated a significant main effect of Age, *F*(1,27) = 27.62, *p* < 0.001, η^2^ = 0.52, as well as a significant main effect of Electrode position, *F*(1,27) = 9.91, *p* < 0.001, η^2^ = 0.28. The Pain × Age interaction was also significant, *F*(1,27) = 5.38, *p* < 0.05, η^2^ = 0.18. The *Post hoc* analyses showed that the Pain effect was only significant in the group of adolescents (see Figure 1). The main effect of Pain and the PainxAge interaction were also significant, *F*(1,27) = 5.38, *p* < 0.05, η^2^ = 0.17.

#### N340

The ANOVA conducted over fronto-central electrodes (FCz, FC3–FC4) showed a significant main effect of Age, *F*(1,27) = 23.93, *p* < 0.001, η^2^ = 0.48, as well as a significant main effect of Electrode position, *F*(1,27) = 28.63, *p* < 0.001, η^2^ = 0.52. The main effect of Pain was also significant, *F*(1,27) = 5.57, *p* < 0.05, η^2^ = 0.18, indicating that painful stimuli generated less negative amplitude than neutral ones. As there were no significant interaction between Age and Pain, this suggests that the effect of Pain was similar for adults and adolescents (see Figure 1).

#### Late positive potential

The ANOVA conducted over parietal electrodes (Pz, P3, and P4) showed a significant main effect of Age, *F*(1,27) = 91.60, *p* < 0.001, η^2^ = 0.78 and Electrode position, *F*(1,27) = 5.31, *p* < 0.01, η^2^ = 0.20. Results also displayed a significant Pain × Task interaction, *F*(1,27) = 7.49, *p* < 0.01, η^2^ = 0.22. *Post hoc* analyses suggested that the Pain effect was significant only in the pain judgment condition, i.e., when attention was directed toward pain indices (Figure 2). The Electrode position × Pain × Task interaction was also significant, *F*(1,27) = 3.37, *p* < 0.05, η^2^ = 0.12. *Post hoc* analyses showed that the Pain × Task interaction was significant only over Pz. Results further showed a significant Task × Age interaction, *F*(1,27) = 5.94, *p* < 0.05, η^2^ = 0.19, indicating that in adolescents amplitudes were higher when they had to judge for pain than when they had to count hands, while no task effect was significant in adults. This suggests that the mere fact of orienting attention toward pain indices generated enhanced amplitudes on this late positive potential.

#### Correlations between brain potentials and behavioral measures

To investigate whether the electrophysiological activity elicited by the painful stimuli was correlated with subjective evaluation of other’s pain and self-unpleasantness, with self-assessed empathic abilities, and with resistance to interference, we computed correlations between the mean amplitudes of ERPs elicited by painful stimuli for each component (N110, N340, and LPP), over electrodes displaying the stronger Pain effect (FCz, for N110 and N340 and Pz for LPP).

In addition, correlations between the mean amplitudes elicited in the pain judgment task in P4 (showing the stronger Task × Age interaction) and behavioral measures (subjective evaluation of other’s pain and the self-unpleasantness, self-assessed empathic abilities, and resistance to interference) were computed to characterize the Age × Task interaction observed over the LPP on the right electrode.

Lastly, correlations between subjective ratings and self-assessed empathic abilities were analyzed.

In order to better describe differences between age-related processes, correlations were assessed for each age group separately (see Table 4).

**Table 4:** **Correlations between ERPs amplitudes and behavioral measures in adults and adolescents**.

	EM	EMC	EMA	INT	Other	Self
**ADOLESCENTS**						
N110 painful	0.36	0.66_c_**	0.41	0.48	−0.02_f_	−0.18
N340 painful	0.15	0.29	0.27	0.42	−0.15	−0.36
LPP painful	−0.21	−0.27	−0.32_e_	−0.19	0.07	−0.09
LPP pain judgment	−0.22	−0.46	−0.37	−0.72***	−0.01	0.18
Other	0.69_a_***	0.50*	0.40			
Self	0.61_b_**	0.38_d_	0.44			
**ADULTS**						
N110 painful	0.32	0.17_c_	0.33	0.38	−0.61_f_**	−0.49*
N340 painful	−0.05	−0.24	0.15	0.27	−0.49*	−0.34
LPP painful	0.05	−0.01	0.35_e_	−0.20	−0.14	0.31
LPP pain judgment	−0.05	0.12	0.02	−0.45	0.43	0.31
Other	−0.08_a_	0.03	−0.20			
Self	−0.13_b_	−0.26_d_	−0.04			

*EM, empathy; EMC, cognitive empathy; EMA, affective empathy; INT, Stroop interference; Other, other’s pain evaluations; Self, self-unpleasantness ratings. Correlations coefficients with same subscripts differ significantly (comparison via Fisher’s *Z* transformation, *p* < 0.07)*.

*p < 0.07.

**p < 0.05.

***p < 0.01.

Analyses carried out on the N110 amplitudes showed, for the adults, high correlations between ERPs’ amplitudes and subjective ratings of other’s pain on the one hand (*r* = −0.61, *p* < 0.05), and subjective ratings of self-unpleasantness on the other hand (*r* = −0.49, *p* = 0.06). While adolescents’ ERP displayed quasi-null correlations with subjective ratings, they were significantly and inversely related to cognitive empathy abilities (*r *= 0.66, *p* < 0.05).

For the N340, correlations between ERPs’ amplitudes and subjective ratings of other’s pain were still high and not far for significance in the group of adults (*r* = −0.49, *p* = 0.06).

Analyses carried out over the LPP amplitudes showed interesting correlations between P4 amplitudes and interference scores (*r* = −0.72, *p* < 0.01) in adolescents.

Results also showed strong positive correlations between empathic abilities and subjective ratings in adolescents, but not in adults.

## Discussion

This study aimed at investigating the neural correlates of affective and cognitive empathy in adolescents compared to that of young adults. This was achieved by constraining attention to or away from pain cues, in order to dissociate the automatic response to other’s pain from the cognitive empathic response. Consistently with previous studies (Fan and Han, 2008; Han et al., 2008; Decety et al., 2010), our results showed the expected responses: an early fronto-central automatic response to others’ pain that was independent of top-down attention to pain cues, and a late parietal cognitive response to pain that was modulated by task demand. Age-related differences in ERPs associated with painful stimuli consisted in, on the one hand, an earlier automatic response to others’ pain in adolescents than in adults and, on the other hand, a task effect on the late cognitive component only in adolescents.

Consistently with previous studies (Fan and Han, 2008; Han et al., 2008; Decety et al., 2010), our results showed a main effect of pain that was independent of top-down attention to pain on early fronto-central components. These early ERPs associated to painful stimuli were positively related to subjective ratings of both self-unpleasantness and judgment of other’s pain in adults, which underlies the affective dimension of early automatic brain response to others’ pain (note that such relation was not observed in adolescents, this will be discussed further). This finding confirms previous assumptions of an automatic emotional sharing component of empathy. Interestingly, our results showed that the pain effect was significant only in adolescents on the N110, and in both adults and adolescents on the N340, suggesting an earlier differentiation between neutral and painful stimuli in adolescents than in adults. It may be assumed that affective stimuli are more salient to adolescents and therefore earlier detected. Some studies have for example shown that merely viewing emotional pictures generated enhanced activity of the amygdala in adolescents as compared to adults or young children (Hare et al., 2008); this may be interpreted as a sign of higher relevance of emotional information in adolescence (Sander et al., 2003). During this especially vulnerable period, it is likely that emotional indices have a particular significance in the growing importance of social interactions. The earlier affective sharing mechanism in adolescence, as compared to young adults, may then be linked to higher motivational tendencies toward social interactions. It may also be imputed to immature cognitive regulation processes (Steinberg, 2008), which would not be efficient enough to down-regulate a heightened automatic affective response. Such a view is consistent with a neurobiological model of competition between enhanced activity in subcortical emotional processing systems and less mature top-down prefrontal systems (Hariri et al., 2002, 2003; Decety and Lamm, 2006).

Contrary to adults, this heightened emotional reactivity to other’s pain was not related to subsequent subjective ratings of both self-unpleasantness and judgments of others’ pain, but rather to self-reported cognitive empathic abilities. Specifically, the lower the cognitive empathic abilities, the greater the brain response to painful stimuli. In adolescents then, the early automatic brain response to observing someone else’s pain seems to be linked to social emotion regulation abilities, and especially to perspective-taking abilities, which the cognitive empathy scale of EQ mostly address. This finding is consistent with prior research reporting a linear increase in social perspective-taking from childhood to adulthood (Selman, 1980; Davis and Franzoi, 1991), and suggests that emotion regulation mechanisms play a crucial role in the adolescent affective empathic response. Theoretical and empirical links have been made between the development of perspective-taking abilities and higher levels of moral reasoning (Kohlberg and Candee, 1984; Eisenberg et al., 2005). Kohlberg and Candee (1984) argued that moral reasoning increases with age because of age-related structural changes in reasoning (i.e., the development of qualitatively new ways of thinking). In their view, as adolescent mature, moral judgment develops as a consequence of advances in perspective-taking abilities. Consistently, in a longitudinal study following mid-adolescents (15 years) until adulthood (26 years), Eisenberg et al. (2005) report a decrease in personal distress and increased perspective-taking and prosocial moral reasoning. In addition, changes in conceptions of the self from childhood into adolescence likely are associated with moral and prosocial development. By late adolescence, the self is defined in terms of social and psychological aspects, with the consequence that morality constitutes a major regulator of social interactions (e.g., Harter, 1999). In line with this literature, our results point to the importance of social cognitive competencies in very automatic aspects of prosocial abilities in early adolescence. More specifically, it may be assumed that the link between an earlier automatic processing of pain, as compared to young adults, and cognitive abilities reflects a developing integration of both affective and cognitive aspects of empathy. Interestingly, self-assessed dispositional empathy was strongly correlated with both ratings of others’ pain and judgment of self-unpleasantness in adolescents only. On the one hand, this observation comforts the idea that the task used in the present experiment calls to empathic processes. On the other hand, the absence of such correlations in adults raises question. It might be assumed that emotional processes are more related in adolescence that in adulthood. A “differentiation hypothesis” has been proposed concerning changes in the functional organization of cognitive abilities during child development (Garrett, 1946). It postulates that the structure of intelligence develops from a relatively unified, general ability in childhood to more differentiated, specific cognitive abilities by early adulthood (see Shing et al., 2010, for recent evidence). As cognitive abilities are thought to become more involved in emotional processes during childhood (Labouvie-Vief et al., 2010, in press), a similar functional reorganization might occurs with emotional processes. This hypothesis however needs further empirical evidence.

Our results also showed a late effect of pain that was modulated by task demand over central parietal areas both in adults and adolescents, i.e., the dissociation between painful and neutral stimuli was observed only when attention was oriented toward pain cues. This finding is consistent with results of a previous fMRI study showing that neural underpinnings of affective and cognitive empathy are already at place in pre-adolescence (Decety et al., 2008). This late parietal component observed in empathy for pain has been proposed to reflect the evaluation process of stimuli showing others in painful situations (Fan and Han, 2008). Accordingly, painful stimuli would require a higher attentional demand than neutral ones in the pain judgment task, thus inducing deeper evaluation of the situation. Interestingly, results showed, over the right parietal region, a main effect of the task in adolescents but not in adults. That is, adolescents displayed enhanced amplitudes when they had to judge other’s pain as compared to the simple counting task. In other words, the mere fact of orienting attention to pain cues induced heightened amplitudes in ERPs reflecting the process of evaluation of the situation. Results further show a correlation between amplitudes of this ERP and the interference level observed in the Stroop task in adolescents: the higher the interference displayed by adolescents, the higher the amplitudes. Thus, it seems that judging others’ pain requires the recruitment of additional resources by adolescents and that this additional activity is linked to lower inhibition abilities. ERP studies on emotion processing suggest that the amplitude of the LPP is mostly determined by emotional arousal (Schupp et al., 2000). Moreover, it has been recently suggested that this ERP represent a relevant neural marker for emotion regulation, the lower the amplitude the better the regulation (Dennis and Hajcak, 2009; Hajcak and Dennis, 2009).

Increased amplitude of the LPP observed in young adolescents when attended to pain may then reflect a lack of emotion regulation abilities, leading to enhanced emotional arousal when attention is drawn to pain cue. This interpretation is in line with earlier reports of lower inhibitory functions involved in self-regulation in early adolescence (Leon-Carrion et al., 2004; Steinberg, 2008). More specifically, the development of empathy as a complex response to someone else’s distress is thought to rely upon the maturation of the fronto-limbic emotion regulation system, although regulation processes involved in social *versus* basic emotions (such as fear) may substantially differ. It may indeed be assumed that the regulation of personal distress underlying mature empathy calls more to emotion understanding mechanisms or theory of mind abilities (ToM; e.g., Singer, 2006) rather than simply to reappraisal processes. In this sense, developmental differences have been reported in affective ToM task performances of adolescents and adults, adolescents making more errors than adults (Sebastian et al., 2012). Furthermore, fMRI studies suggest that the neural substrates of ToM continue to develop during adolescence, long after children are able to perform complex cognitive and affective ToM tasks (see Blakemore, 2008 for a review).

One limitation of our study is that participants are only women. Nevertheless, gender differences in empathy are well documented in the literature (e.g., Davis, 1980; Eisenberg and Fabes, 1998; Han et al., 2008; Yang et al., 2009). Most research indeed report higher scores of empathy in women. Furthermore, using a similar paradigm than the one we used, Han et al. (2008) showed that women displayed an enhanced effect of pain over the late cognitive component of empathy in comparison to men, suggesting that women intend to undergo more intensive evaluation of painful stimuli. Gender differences have also been reported in the development of prosocial competencies during adolescence (Eisenberg and Fabes, 1998; Eisenberg et al., 2005). Eisenberg and collaborators have for example shown that gender differences in empathic and moral reasoning abilities increased from adolescence through adulthood, which according to the authors is related to increased emphasis on gender-related norm. Future studies will therefore be necessary to better investigate the integration of affective and cognitive aspects of empathy during adolescence.

With this limitation in mind, our results point to the importance of self-regulation abilities in the development of social emotion like empathy during adolescents. Moreover, they are consistent with the idea of continuously developing interrelations between cognitive and emotional processes in childhood and adolescence (Lewis, 2007; Lewis et al., 2010).

## Conflict of Interest Statement

The authors declare that the research was conducted in the absence of any commercial or financial relationships that could be construed as a potential conflict of interest.
